# A randomized trial predicting response to cognitive rehabilitation in multiple sclerosis: Is there a window of opportunity?

**DOI:** 10.1177/13524585221103134

**Published:** 2022-06-28

**Authors:** Stefanos E Prouskas, Menno M Schoonheim, Marijn Huiskamp, Martijn D Steenwijk, Karin Gehring, Frederik Barkhof, Brigit A de Jong, Margriet M Sitskoorn, Jeroen JG Geurts, Hanneke E Hulst

**Affiliations:** Department of Anatomy and Neurosciences, MS Centre Amsterdam, Amsterdam Neuroscience, Amsterdam UMC, Vrije Universiteit Amsterdam, Amsterdam, The Netherlands; Department of Anatomy and Neurosciences, MS Centre Amsterdam, Amsterdam Neuroscience, Amsterdam UMC, Vrije Universiteit Amsterdam, Amsterdam, The Netherlands; Department of Anatomy and Neurosciences, MS Centre Amsterdam, Amsterdam Neuroscience, Amsterdam UMC, Vrije Universiteit Amsterdam, Amsterdam, The Netherlands; Department of Anatomy and Neurosciences, MS Centre Amsterdam, Amsterdam Neuroscience, Amsterdam UMC, Vrije Universiteit Amsterdam, Amsterdam, The Netherlands; Department of Cognitive Neuropsychology, Tilburg University, Tilburg, The Netherlands/Department of Neurosurgery, Elisabeth-TweeSteden Hospital, Tilburg, The Netherlands; Department of Radiology and Nuclear Medicine, Amsterdam Neuroscience, Amsterdam UMC, Vrije Universiteit Amsterdam, Amsterdam, The Netherlands/Institutes of Neurology and Healthcare Engineering, UCL, London, UK; Department of Neurology, MS Centre Amsterdam, Amsterdam Neuroscience, Amsterdam UMC, Vrije Universiteit Amsterdam, Amsterdam, The Netherlands; Department of Cognitive Neuropsychology, Tilburg University, Tilburg, The Netherlands; Department of Anatomy and Neurosciences, MS Centre Amsterdam, Amsterdam Neuroscience, Amsterdam UMC, Vrije Universiteit Amsterdam, Amsterdam, The Netherlands; Department of Anatomy and Neurosciences, MS Centre Amsterdam, Amsterdam Neuroscience, Amsterdam UMC, Vrije Universiteit Amsterdam, Amsterdam, The Netherlands

**Keywords:** Functional MRI, multiple sclerosis, rehabilitation, resting-state, quantitative MRI, treatment response

## Abstract

**Background:**

Cognitive training elicits mild-to-moderate improvements in cognitive functioning in people with multiple sclerosis (PwMS), although response heterogeneity limits overall effectiveness.

**Objective:**

To identify patient characteristics associated with response and non-response to cognitive training.

**Methods:**

Eighty-two PwMS were randomized into a 7-week attention training (*n* = 58, age = 48.4 ± 10.2 years) or a waiting-list control group (*n* = 24, age = 48.5 ± 9.4 years). Structural and functional magnetic resonance imaging (MRI) was obtained at baseline and post-intervention. Twenty-one healthy controls (HCs, age = 50.27 ± 10.15 years) were included at baseline. Responders were defined with a reliable change index of 1.64 on at least 2/6 cognitive domains. General linear models and logistic regression were applied.

**Results:**

Responders (*n* = 36) and non-responders (*n* = 22) did not differ on demographics, clinical variables and baseline cognition and structural MRI. However, non-responders exhibited a higher baseline functional connectivity (FC) between the default-mode network (DMN) and the ventral attention network (VAN), compared with responders (*p* = 0.018) and HCs (*p* = 0.001). Conversely, responders exhibited no significant baseline differences in FC compared with HCs. Response to cognitive training was predicted by lower DMN-VAN FC (*p* = 0.004) and DMN-frontoparietal FC (*p* = 0.029) (Nagelkerke *R*^2^ = 0.25).

**Conclusion:**

An intact pre-intervention FC is associated with cognitive training responsivity in pwMS, suggesting a window of opportunity for successful cognitive interventions.

## Introduction

Multiple sclerosis (MS) is the most frequent demyelinating, inﬂammatory and neurodegenerative disease of the central nervous system (CNS) in young adults. Next to the well-known physical symptoms of the disease, cognitive impairment is present in up to 65% of the people with multiple sclerosis (PwMS).^1^ Cognitive decline is characterized by a slowed information processing speed, impaired memory function as well as problems with attention and executive function, causing (severe) problems in patient’s daily lives (e.g. unemployment).^1,2^

Given the prevalence and impact of cognitive dysfunction in PwMS, effective cognitive training strategies are urgently needed. Restorative, non-pharmacologic interventions, especially computerized cognitive training, show potential for improvements in cognitive functioning in PwMS,^3,4^ although these improvements are at best mild-to-moderate on a group level (effect sizes ranging from 0.06 to 0.23 standardized mean difference).^3^

The high variability in individual response to cognitive training (i.e. substantial improvements in some patients and no improvement or even decline in others) limits the *overall* group effect of restorative interventions.^5^ This might hamper our enthusiasm for such interventions, obscuring the fact that for a subset of patients these interventions may be highly beneficial. This introduces a challenge to identify, beforehand, which patients will benefit from such interventions and which patients will not.

So far, only a few exploratory studies have hinted towards specific patient characteristics being beneficial for cognitive training, that is having a high likelihood of successful treatment response. For example, less grey and white matter atrophy, a relapsing-remitting disease course, and higher processing speed were predictive of better response to training,^6,7^ as were different profiles of default-mode network connectivity.^8^

In this study, we investigated the effect of a restorative computerized cognitive training programme (attention training) on cognition in a large sample of PwMS. Next, we compared baseline demographic, clinical and magnetic resonance imaging (MRI) characteristics between responders and non-responders to identify clinical and MRI-based characteristics of response (and non-response).

## Materials and methods

This study was approved by the Medical Ethical Committee of the VUmc. Informed consent was obtained prior to participation.

### Participants

**Figure 1.**
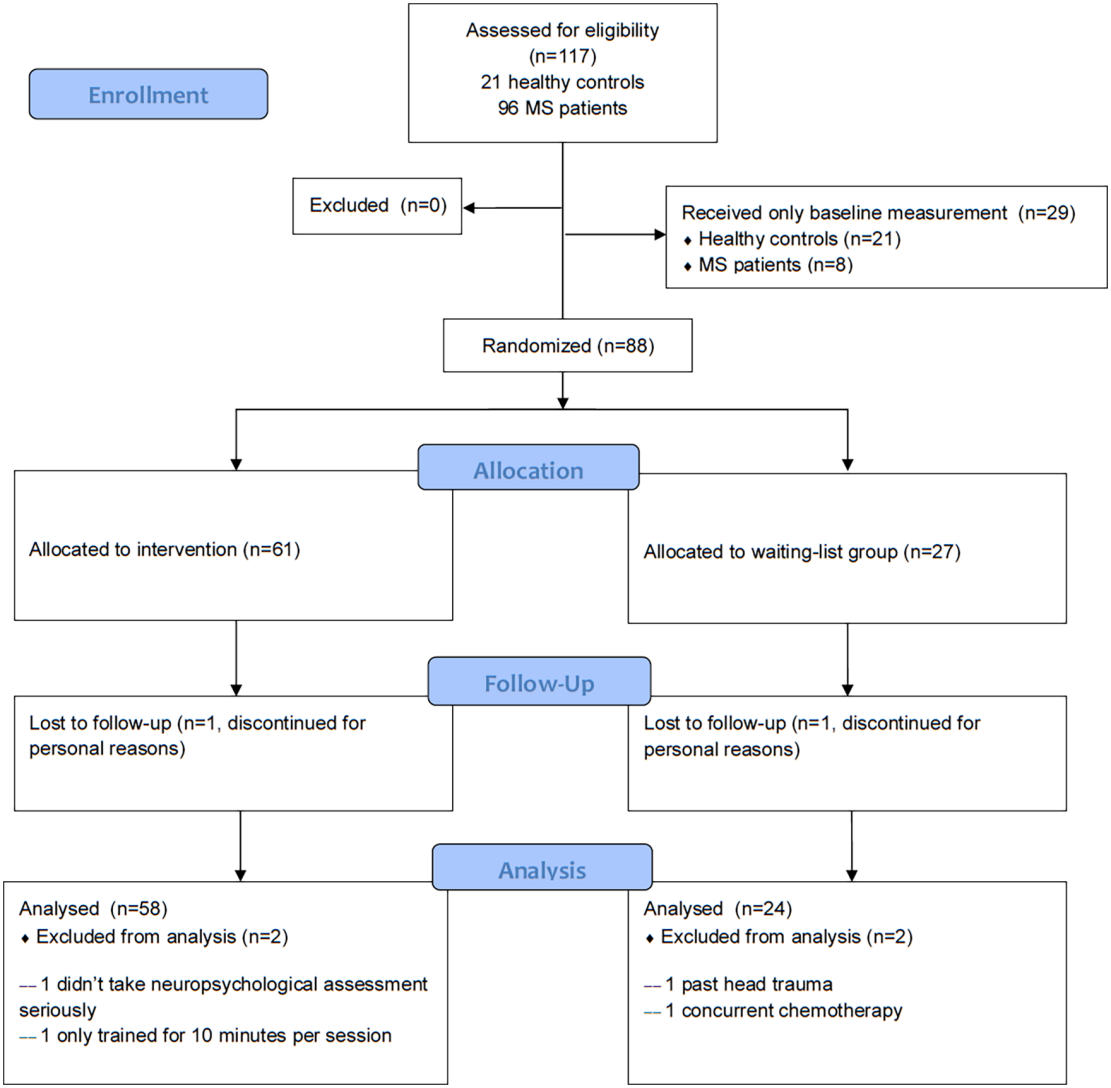
Flow diagram of patient inclusion.

Eighty-two PwMS were included. Inclusion criteria were: MS diagnosis according to the McDonald criteria,^9^ 18–68 years of age, and ability to safely undergo an MRI examination. Patients were screened for motor and visual skills to ensure cognitive training participation (Figure 1). Exclusion criteria included drug abuse, neurological and psychiatric diseases, prior cognitive training participation, and relapses or steroid use 4 weeks prior to examination. To assess disease severity, a validated Expanded Disability Status Scale (EDSS)-based questionnaire was used.^10^ Patients underwent visual and motor screening to ensure intervention participation. Patients were randomized (by means of computer-generated tables) into an intervention group (*n* = 58) or a waiting-list control group (*n* = 24). Treatment allocation was not concealed, and there was no blinding. Twenty-one age-, sex- and education-matched healthy controls (HCs) were included at baseline.

### Intervention

The intervention consisted of the C-Car computer programme, previously used in the field of neuro-oncology.^11^ C-Car combined with compensatory training has been effective in a similarly sized glioma patient sample.^11^ Glioma patients and PwMS exhibit comparable, mostly subtle cognitive impairment. The focus on attention is based on the fact that attention is key for proper functioning in other cognitive domains such as memory and information processing speed. In MS, deficits in attention have been linked to deficits both in working memory and processing speed,^12^ making C-Car an interesting programme for this study.

**Figure 2.**
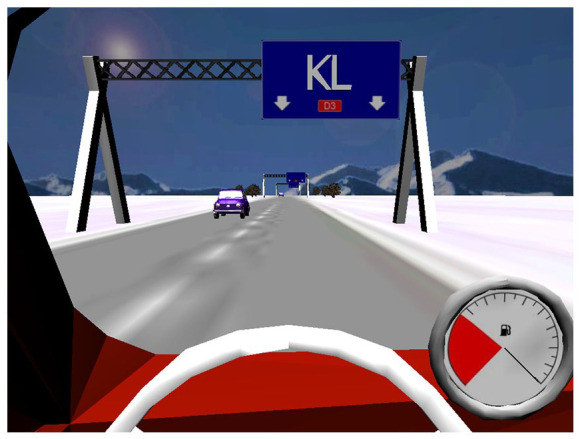
Example of the C-Car computer programme interface. In this particular task, participants are required to form words out of two consecutive road signs (which each present two letters), while at the same time pay attention to the petrol gauge that gradually empties.

C-Car simulates driving a car, with tasks designed to train sustained, selective, alternating and divided attention. C-Car simulates driving a car while presenting information processing tasks. Tasks include forming words out of two consecutive road signs (which each present two letters), counting the number of letters in a word, performing basic arithmetic operations (addition and subtraction), and ranking words in alphabetical order (Figure 2). With increasing difficulty, distractions are added: distracting noise to ignore, and a moving pointer of the petrol gauge to which attention should also be paid. The programme is adaptive; patients practice at their own level, and difficulty is increased throughout the sessions (e.g. faster stimulus rate and longer exercise duration, addition of aforementioned distractions). A score of at least 90% is needed to progress to the next difficulty level. Patients were provided with a laptop, and were required to train for 7 weeks (once a week, 45 minutes per session) at home. Researchers kept weekly contact with patients to ensure compliance (defined as the total time spent training being 75% or more).

### Neuropsychological assessment and questionnaires

All patients underwent neuropsychological assessment at baseline (*T*_0_), post-intervention (*T*_1_) and 3 months follow-up (*T*_2_). HC underwent neuropsychological assessment only at baseline (*T*_0_). The following six cognitive domains, relevant to MS-specific cognitive decline,^4^ were measured:

- Verbal memory (California Verbal Learning Test – II; CVLT^13^)
- Information processing speed (Oral Letter Digit Substitution Test; LDST^14^)
- Visuospatial memory (Location Learning Test; LLT^15^)
- Working memory (WAIS-III Digit Span; DS and WAIS-III Letter Number Sequencing test; LNS^16^)
- Verbal fluency (Word List Generation; WLG^14^)
- Attention (Stroop,^17^ Concept Shifting Test; CST,^18^ D2-test,^19^ and Test of Everyday Attention; TEA^20^)

To reduce learning effects, parallel test versions were used where available (CVLT and TEA). The primary outcome was a statistically significant improvement in attention (as measured with the tests mentioned above) in the intervention group compared with the waiting-list group. Average cognition was assessed by calculating the mean of *z*-scores (based on the HC group) of all cognitive domains.

Questionnaires were administered to measure anxiety and depression (Hospital Anxiety and Depression Scale),^21^ fatigue (Checklist Individual Strength 20-revised),^22^ and subjective cognitive complaints (Cognitive Failures Questionnaire^23^).

### Defining response

To differentiate responders from non-responders, a reliable change index (RCI) from *T*_0_ to *T*_1_ was calculated for each cognitive test score.^24^ To correct for practice effects, scores of the waiting-list control group were used to calculate the standard error of difference. If patients reached a post-intervention change in test score which corresponded to an RCI threshold of 1.64 (90% confidence interval), a reliable improvement (>+1.64) or decline (<−1.64) was designated. Responders were defined as scoring above the RCI threshold on at least two out of six (33%) of the cognitive domains measured (see above), on at least one test per domain.

### MRI protocol

All patients underwent brain MRI scanning at baseline (*T*_0_) and post-intervention (*T*_1_). HCs underwent MRI scanning only at baseline (*T*_0_). All subjects were scanned on a 1.5 T whole-body magnetic resonance system (Siemens Magnetom Avanto Syngo, Erlangen, Germany), using an eight-channel phased-array head coil). The details of the MRI protocol are described in the Supplementary Material.

### Grey matter, white matter and lesion volumes

White matter lesions were automatically segmented on the PD/T2 images using *k*-nearest neighbour classification with tissue-type priors,^25^ which was also used to compute lesion volume. The lesion segmentations were visually inspected and manually corrected where necessary. Subsequently, white matter lesion masks were registered to the 3D T1-weighted images to enable lesion filling.^26^ Whole-brain, grey and white matter volumes were calculated on the lesion-filled images using SIENAX, following previously published pipelines^27^ and deep grey matter volumes were obtained using FIRST.

### Diffusion-weighted imaging processing

Diffusion tensor imaging (DTI) data were pre-processed using motion and eddy current correction on images and gradient vectors followed by diffusion tensor fitting (in FMRIB Software Library FSL). To assess white matter integrity, fractional anisotropy (FA) maps were computed and non-linearly registered to the FMRIB58_FA brain. Next, FA maps were averaged across subjects and skeletonized to obtain the main white matter tracts common to the entire sample using the standard Tract-Based Spatial Statistics pipeline (part of FSL).

To obtain individual measures of whole-brain white matter integrity damage, the severity and extent of white matter damage was quantified based on FA (see Supplementary Material).

### Resting-state functional connectivity

**Figure 3.**
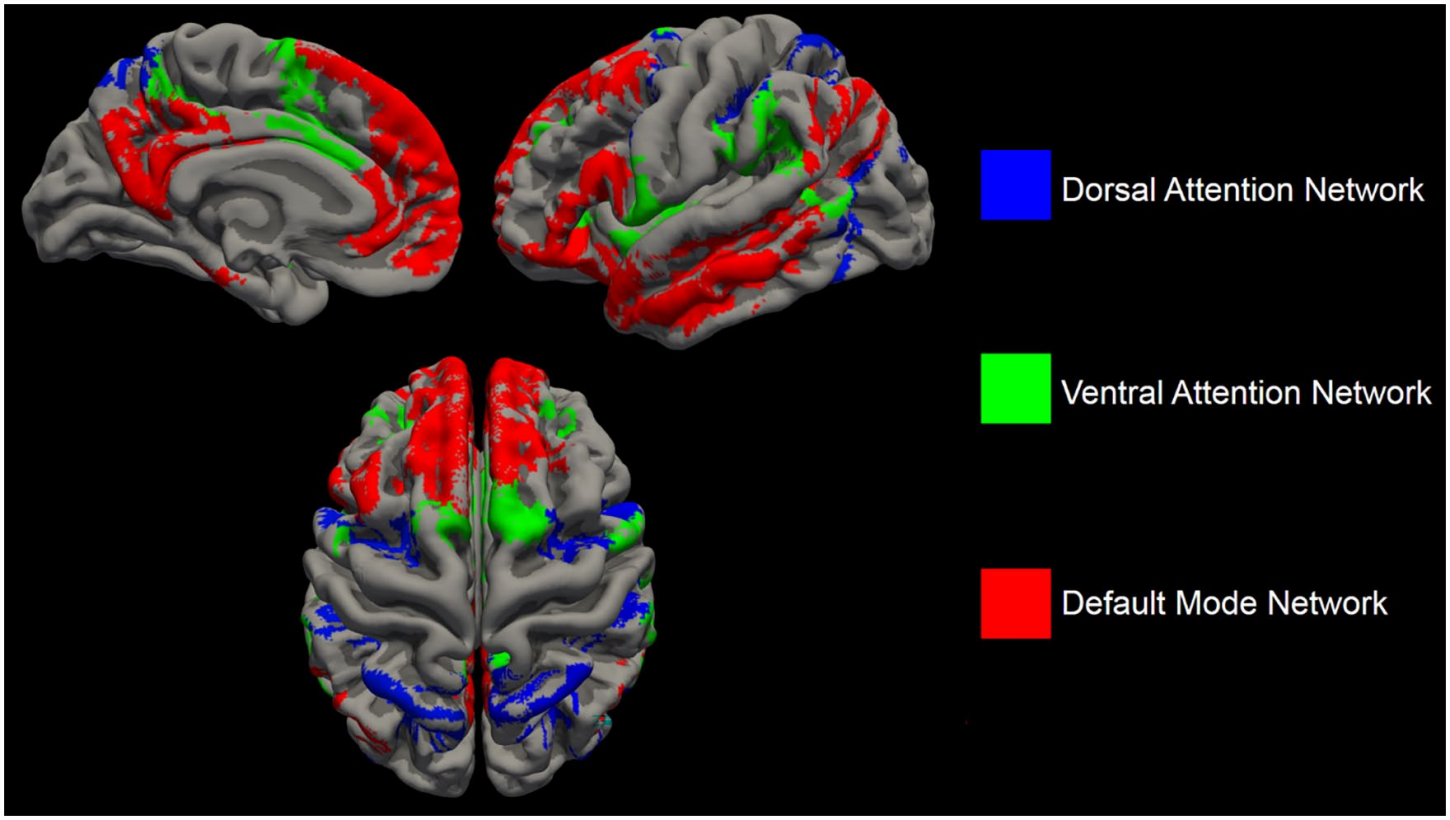
The cortical areas comprising the dorsal attention network (blue), ventral attention network (green) and default-mode network (red).

To define which resting-state network each region belonged to, the Yeo resting-state network atlas^28^ was overlaid on the Brainnetome atlas,^29^ after which each region was defined to one network based on maximal overlap. Based on previous literature on attention and FC,^30^ networks relevant to attention were chosen, that is the dorsal attention network (DAN), ventral attention network (VAN), frontoparietal network (FPN) and default-mode network (DMN) (Figure 3). See also Supplementary Material.

### Statistical analyses

Statistical analyses were performed in SPSS, version 26. Independent samples *t*-tests and chi-square tests were used to assess baseline group differences, and nonparametric tests were used for variables that were non-normally distributed. To assess the effects of the intervention, linear mixed models were used with the intercept as a random factor, and group (intervention vs. waiting-list) and time (*T*_0_ vs. *T*_1_ vs. *T*_2_) as fixed factors. After the intervention group was divided into responders and non-responders, a multivariate general linear model was used to assess baseline differences in (1) demographic variables, (2) cognitive test scores, (3) structural MRI outcomes and (4) functional MRI outcomes. Finally, a backward logistic regression was performed, with responder/non-responder classification as dependent outcome, to identify the strongest independent baseline predictors of response. The Bonferroni post hoc test was used to examine differences between groups. Variables were entered in four blocks in the following order: demographics (age, sex, level of education, EDSS score), volumetric measures (cortical gray matter volume (GMV)), DTI measures (severity of microstructural white matter damage)and FC measures between the DMN and FPN, DAN and VAN. Group comparison *p*-values < 0.05 after correcting for multiple comparisons (Bonferroni correction) were considered statistically significant.

## Results

### Baseline characteristics

The sample consisted of 82 PwMS (53 female, mean age 48.46 (9.92) years, mean disease duration 13.52 (8.24) years, median EDSS = 4.0, 1 CIS, 58 RRMS, 20 SPMS, 3 unknown disease course) and 21 HC (15 female, mean age 50.27 (10.15) years) (Table 1). Patients in the intervention group trained, on average, for 138.60 minutes (*SD* = 54.20), that is 77.87% of the total programme duration.

**Table 1.** Demographics and brain volumes of the entire sample..

	Healthy controls, (*n* = 21)	MS, (*n* = 82)	Waiting-list, (*n* = 24)	Intervention, (*n* = 58)	Responders, (*n* = 22)	Non-responders, (*n* = 36)
Demographics						
Age, years	50.27 (10.15)	48.46 (9.92)	48.51 (9.37)	48.44 (10.21)	47.48 (8.98)	49.02 (10.98)
Female, *n* (%)	15 (71.4)	53 (64.6)	19 (79.2)	34 (58.6)	14 (63.6)	20 (55.6)
Level of education^a^	6 (4–7)	6 (3–7)	6 (5–7)	6 (3–7)	6 (5–7)	6 (3–7)
Disease duration, years		13.52 (8.24)	13.10 (9.6)	13.69 (7.73)	14.24 (8.47)	13.35 (7.34)
EDSS^a^		4 (0–7.5)	4 (2.5–7.5)	4 (0–7.5)	4 (2.5–7.5)	4 (0–7)
Disease phenotype (CIS/RRMS/SPMS/unknown)		1/58/20/3	0/16/6/2	1/42/14/1	0/15/6/1	1/27/8/0
% Cognitively impaired patients^b^		31.7%	33.3%	31.0%	35.7%	29.6%
Normalized brain volumes						
Cortical grey matter volume, ml	800.1 ± 39.4*	736.4 (69.4)*	749.0 (65.4)	731.3 (70.8)	717.0 (53.6)	739.9 (79.0)
White matter volume, ml	764.4 (38.9)*	734.1 (36.8)*	735.5 (36.9)	733.5 (37.0)	736.8 (38.2)	731.5 (36.7)
Deep grey matter volume, ml	62.6 (5.8)*	56.6 (10.1)*	55.7 (9.3)	56.9 (10.5)	57.8 (95)	55.9 (10.4)
Lesion volume, ml		10.2 (10.9)	8.0 (8.4)	11.0 (11.7)	13.1 (13.5)	9.8 (10.5)

MS: multiple sclerosis; EDSS: Expanded Disability Status Scale; CIS: clinically isolated syndrome; RRMS: relapsing-remitting multiple sclerosis; SPMS: secondary-progressive multiple sclerosis.

Baseline demographic, clinical and MRI characteristics. All values represent means and SD, unless otherwise denoted.

a Median and range.

b Defined as having a *z*-score of <−2.0 SD on at least 2 tests.

* Significant difference between patients with multiple sclerosis and healthy controls at *p* < 0.05. No significant differences between responders and non-responders were found.

#### Baseline differences between PwMS and HC

There were no significant differences in age, sex and education between HC and PwMS (Table 1). PwMS had significantly worse average cognition (*t* = −4.41, *p* < 0.001), reported higher levels of depression, anxiety, fatigue, subjective cognitive complaints, and had a more passive coping style on average compared with HC (see Table 2). Compared with HCs, PwMS had significantly less grey matter (GM), white matter (WM) and deep grey matter (DGM) volume, as well as significantly higher FC between DMN-DAN (*p* = 0.012), DMN-VAN (*p* = 0.011) and DMN-FPN (*p* = 0.03) (Table 1).

**Table 2.** Raw scores on neuropsychological evaluation and questionnaires.

	Healthy controls, (*n* = 21)	Waiting-list, (*n* = 24)			Intervention, (*n* = 58)		
		*T*_0_	*T*_1_	*T*_2_	T_0_	T_1_	T_2_
Cognition							
California Verbal Learning Test	67.24 (7.65)*	56.33 (13.07)	58.17 (13.62)^†^	61.64 (15.19)^†^	56.48 (11.5)	59.57 (11.94)^†^	60.4 (12.69)
Letter Digit Substitution Test	64.43 (11.64)*	55.17 (15.08)	57.63 (15.52)^†^	58.05 (16.1)^†^	55.26 (11.69)	58.02 (13.37)^†^	58.58 (15.38)
Digit Span Forward	9.71 (1.98)*	9.04 (1.78)	8.96 (1.83)	9.09 (1.6)	8.52 (2.19)	9.14 (2.09)	9.28 (2.11)
Digit Span Backward	7.86 (1.28)*	6.63 (1w.71)	6.96 (2.2)	7.36 (2.17)^†^	6.71 (1.84)	7.31 (1.89)^†^	7.05 (2.33)
Number Letter Sequencing	11.24 (1.87)	10.96 (2.65)	10.71 (3.48)	11.09 (3.66)	10.28 (2.69)	11.10 (2.93)^‡^	10.42 (3.44)
Word List Generation	17.45 (4.53)*	15.46 (4.21)	16.57 (4.06)^†^	17.07 (4.06)^†^	15.18 (4.07)	16.43 (4.43)^†^	16.63 (4.07)
Location Learning Test^a^	15.1 (10.77)	22.75 (26.93)	19.75 (21.93)	15.41 (20.03)	21.74 (24.54)	15.53 (17.35)	13.56 (20.74)
Stroop^a^	25.86 (7.55)	28.59 (13.9)	26.52 (16.47)	22.72 (16.73)^‡^	30.74 (15.34)	27.47 (12.96)	27.34 (12.5)
D2-test	156.43 (43.44)*	135.17 (59.25)	153.65 (61.33)^†^	154.9 (70.7)	126.81 (40.17)	140.07 (43.13)^†^	152.16 (48.71)^†^
Concept Shifting Test^a^	8.8 (4.84)*	11.69 (14.82)	11.72 (8.28)	11.63 (13.95)	12.95 (13.23)	9.74 (6.36)	10.26 (8.07)
Test of Everyday Attention^b^	–0.01 (0.60)*	–0.64 (1.23)	–0.12 (0.83)^†^	–0.38 (1.26)	–0.70 (1.02)	–0.35 (0.94)^†^	–0.42 (1.0)
Questionnaires							
HADS depression^a^	2.4 (2.98)*	5.21 (3.78)	5.59 (4.25)	6.4 (4.49)	5.93 (3.73)	5.02 (3.41)	6.62 (3.59)
HADS anxiety^a^	4.8 (4.19)*	8.04 (4.63)	7.55 (4.53)	7.65 (4.52)	7.67 (4.1)	6.62 (4.02)	7.82 (4.01)
CIS20-*r* (fatigue)^a^	50.85 (25.23)*	81.13 (23.53)	81.27 (25.98)	93.05 (20.06)	82.82 (19.65)	77.56 (20.11)	79.32 (18.38)^‡^
AIS (sleeping difficulty)^a^	2.89 (3.13)*	5.54 (3.19)	5.59 (4.23)	6.9 (3.54)	5.29 (3.82)	5.15 (4.19)	4.79 (3.66)
Coping styles							
Active	19.6 (2.74)	19.78 (4.72)	20.23 (4.48)	19.37 (4.34)	19.41 (4.21)	19.4 (4.11)	19.1 (4.0)
Seeking distraction	17.8 (3.16)	18.04 (4.31)	17.91 (3.79)	18.37 (3.74)	17.59 (3.96)	17.04 (3.95)	16.54 (3.25)
Avoiding	16.35 (2.76)	17.96 (4.34)	18.48 (4.43)	17.32 (3.35)	17.05 (3.48)	16.62 (3.59)	16.9 (3.65)
Social support	15.1 (3.46)	13.25 (4.15)	13.95 (4.21)	12.58 (4.67)	13.29 (3.72)	13.2 (3.47)	13.31 (3.86)
Passive coping	10.4 (2.87)*	12.63 (4.05)	12.33 (4.81)	13.16 (3.64)	12.03 (3.07)	11.89 (3.03)	11.69 (3.39)
Expressing emotions	5.55 (0.76)	6.38 (1.53)	6.5 (1.65)	6.32 (1.42)	5.83 (1.67)	5.35 (1.81)	5.18 (1.55)
Reassuring thoughts	12.45 (1.93)	13.54 (3.23)	13.09 (3.19)	11.95 (3.31)	12.59 (2.93)	12.44 (3.05)	12.59 (2.91)
Subjective cognition							
Cognitive Failures Questionnaire^a^	21.35 (11.86)*	44.7 (22.64)	43.91 (20.14)	47.84 (20.07)	41.89 (18.08)	38.28 (16.15)	36.36 (15.14)
Cognitive Functioning Scale^a^	5 (4.26)*	13.58 (7.45)	12.82 (7.39)	13.55 (6.48)	11.9 (5.35)	11.13 (5.3)	11.54 (7.85)

HADS: Hospital Anxiety and Depression Scale; AIS: Athens Insomnia Scale; CIS20-r: Checklist Individual Strength – Revised.

Raw scores on cognitive assessment battery and questionnaires. All values represent means and SD, unless otherwise denoted.

a Higher scores indicate worse outcomes.

b *z*-Score.

Significant differences in bold.

* Significant difference between patients with multiple sclerosis and healthy controls at *p* < 0.05.

† Significant time effect for *T*_0_–*T*_1_ at *p* < 0.05.

‡ Significant time group × time effect at *p* < 0.05.

PwMS in the intervention group and in the waiting-list control condition did not differ in age, sex, educational level, cognitive performance and self-perceived cognition and levels of fatigue, anxiety and depression at baseline. Imaging measures were not different between the two groups (Tables 1–3).

### Effects of C-Car

The pre- and post-intervention differences investigated on a group level on short- and long-term follow-up neuropsychological testing are presented here.

#### Immediate effects

**Figure 4.**
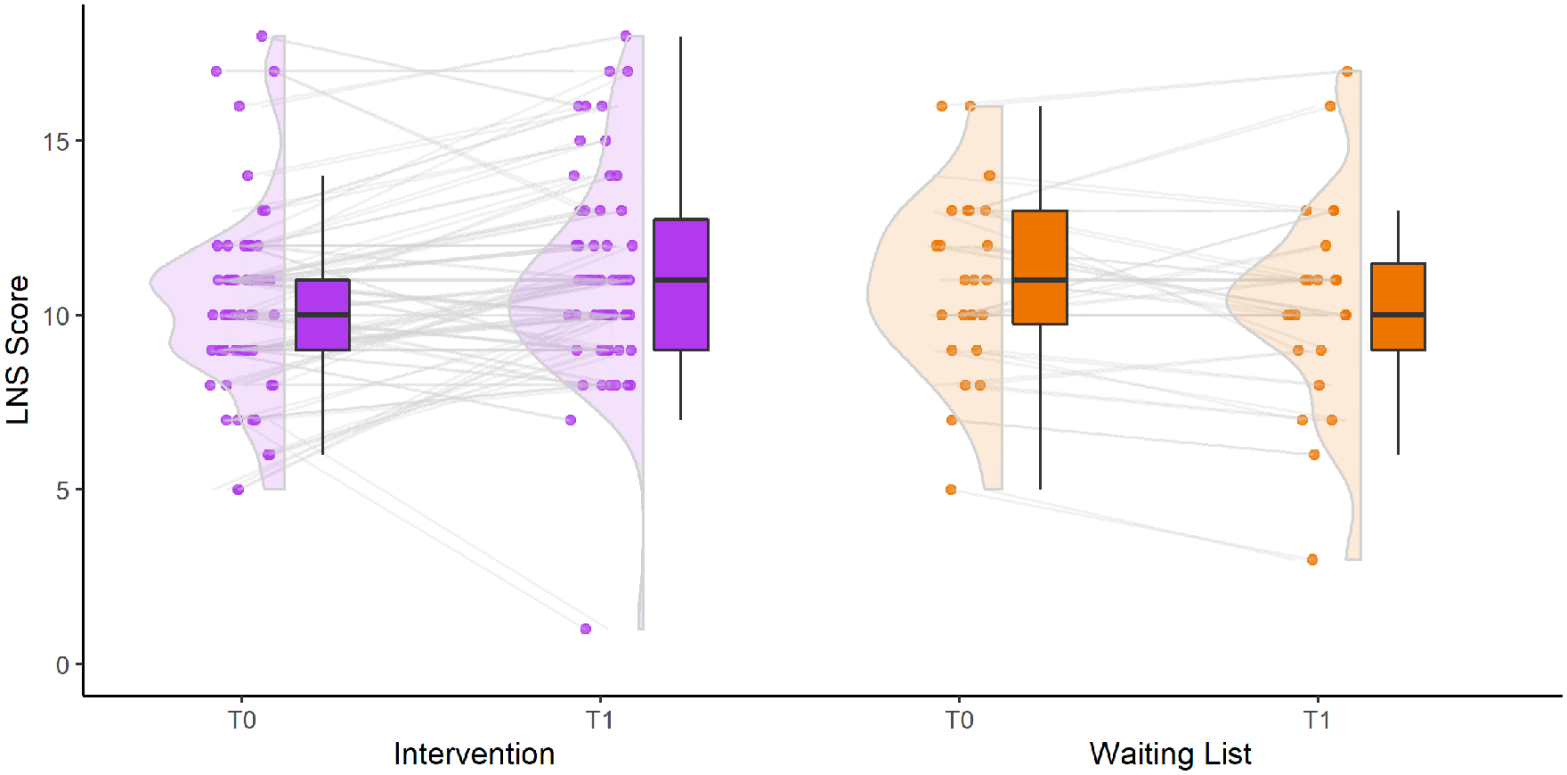
Change in working memory (measured with Letter-Number-Sequencing test) between baseline and post-intervention. Boxplots show median and quartiles, dots show individual scores, with lines connecting individual scores between timepoints.

A significant group × time effect was found for working memory (Letter-Number-Sequencing) between *T*_0_ and *T*_1_ (Figure 4). The intervention group improved (*T*_0_: 10.28 (*SD* = 2.69) versus *T*_1_: 11.1 (*SD* = 2.93)) compared to the waiting-list controls (*T*_0_: 10.96 (*SD* = 2.65) versus *T*_1_: 10.71 (*SD* = 3.48), *F* = 4.470, *p* = 0.038), with a standardized mean difference of 0.37 (effect size). No other significant effects were found for any of the other cognitive tests and questionnaires (Table 2), nor for measures of white matter damage and FC (Table 3).

**Table 3.** Microstructural white matter changes and functional connectivity in the intervention group, waiting-list group, and healthy controls.

	Healthy controls, (*n* = 21)	Waiting-list, (*n* = 24)		Intervention, (*n* = 58)	
		*T*_0_	*T*_1_	*T*_0_	*T*_1_
White matter damage					
Severity^a^	0.0 (0.23)*	–0.23 (0.34)	–0.25 (0.37)	–0.27 (0.34)	–0.27 (0.37)
Extent (number of voxels exceeding *z* = −3.1)	6.67 (14.82)*	1358.3 (1921.16)	1755.8 (3355.0)	1459.3 (2256.2)	1509.1 (2538.9)
Normalized functional connectivity between networks					
Default mode – dorsal attention	0.81 (0.14)*	0.89 (0.16)	0.87 (0.14)	0.89 (0.14)	0.89 (0.14)
Default mode – ventral attention	0.81 (0.13)*	0.87 (0.14)	0.86 (0.15)	0.93 (0.18)	0.93 (0.15)
Default mode – frontoparietal	1.05 (0.22)*	1.14 (0.28)	1.14 (0.24)	1.18 (0.24)	1.16 (0.25)
Dorsal attention – ventral attention	1.21 (0.23)	1.12 (0.25)	1.16 (0.23)	1.17 (0.26)	1.14 (0.22)
Dorsal attention – frontoparietal	1.11 (0.25)	1.19 (0.28)	1.17 (0.25)	1.21 (0.25)	1.17 (0.28)
Ventral attention – frontoparietal	0.94 (0.16)	0.98 (0.19)	1.0 (0.17)	1.04 (0.18)	1.0 (0.17)

Measures of DTI-based white matter damage, resting-state fMRI functional connectivity.

* Significant difference between patients with multiple sclerosis and healthy controls at *p* < 0.05.

#### Three-month follow-up effects

For group differences between *T*_0_, *T*_1_ and *T*_2_, no significant effects were found for any of the cognitive measures. A significant group × time effect was found for fatigue (CIS-20r, *F* = 5.177, *p* = 0.007), with patients in the waiting-list control group experiencing increased fatigue at 3 months follow-up compared with patients in the intervention group (intervention: *T*_0_ = 81.13 (*SD* = 23.53), *T*_1_ = 81.27 (*SD* = 25.98), and *T*_2_ = 93.05 (*SD* = 20.06) versus waiting-list: *T*_0_ = 82.82 (*SD* = 19.65), *T*_1_ = 77.56 (*SD* = 20.11) and *T*_2_ = 79.32 (*SD* = 18.38)). No significant effects were found for any of the other questionnaires, nor for the MRI measures.

### Baseline differences between responders and non-responders

#### Cognitive characteristics

Patients in the intervention group were divided into responders (*n* *=* 22; 38%) and non-responders (*n* *=* 36, 62%) based on immediate post-intervention improvement compared with baseline. Training duration did not significantly differ between responders and non-responders (131.31 (*SD* = 58.24) vs. 142.11 (*SD* = 52.93), *p* = 0.562). Two responders (9.1%) improved on four out of six cognitive domains, seven responders (31.9%) improved on three out of six cognitive domains, and 13 responders (59%) improved on two out of six cognitive domains. Of the cognitive domains for which improvement was found, 19 patients (84.6%) improved on one or more measures of working memory (Digit Span Forward and Backward, Letter-Number-Sequencing), 11 patients (50%) improved on verbal memory (CVLT-II), 10 patients (45.5%) improved on information processing speed (LDST), six patients (27.3%) improved on verbal fluency (WLG), six patients (31.8%) improved on measures of attention (Stroop, D2-test, CST, TEA), and one patient (4.5%) improved on visuospatial memory (LLT). For the non-responders, 18 patients (50%) improved on one cognitive domain, and the remaining 18 did not improve on any cognitive domain.

#### Clinical characteristics

Responders and non-responders did not differ on demographic variables (age, sex, level of education), clinical variables (disease duration, EDSS score, disease phenotype), baseline cognition, and any of the questionnaires (depression, anxiety, fatigue, sleeping difficulty, coping style, subjective cognition).

#### Imaging

**Figure 5.**
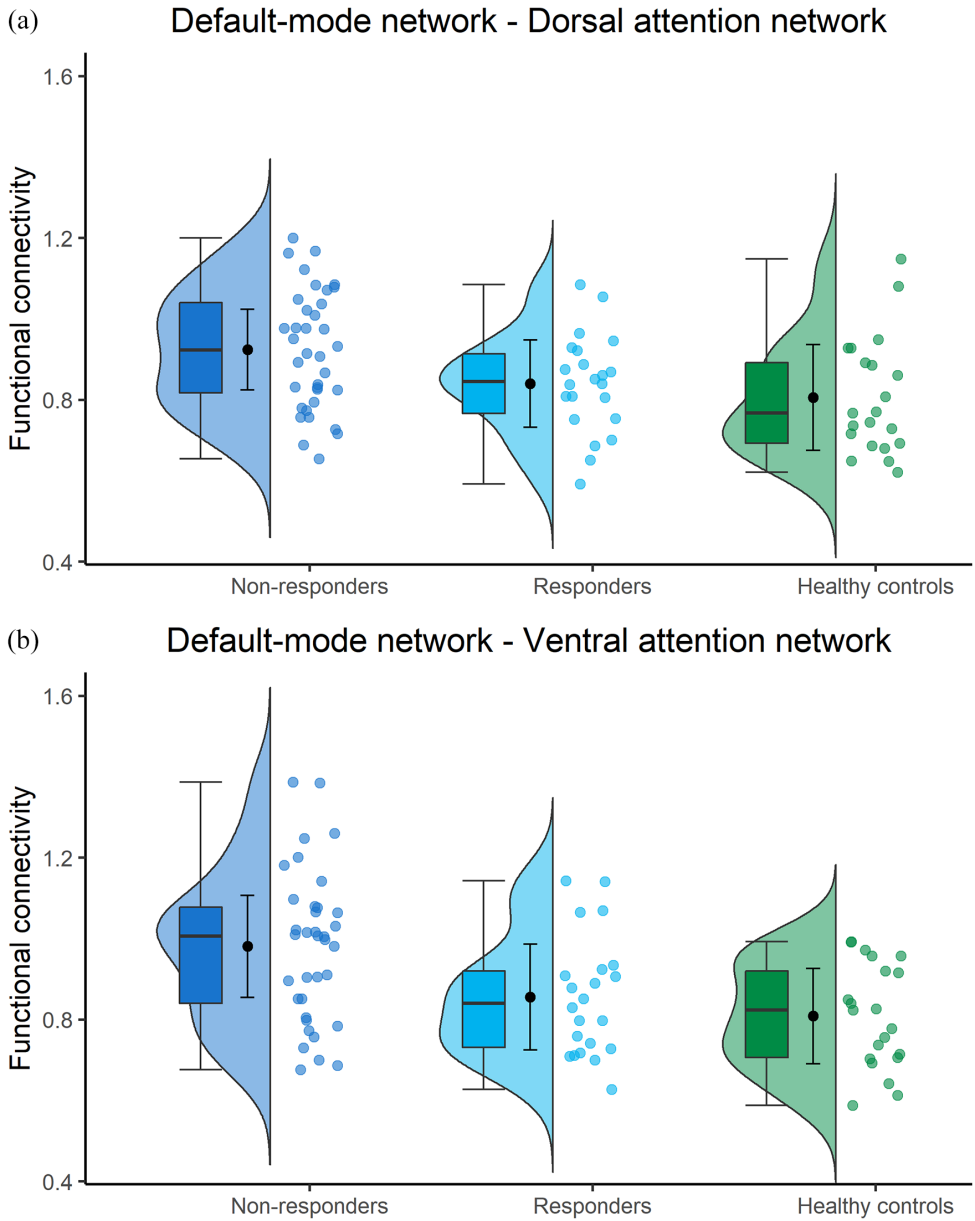
(a) Functional connectivity between the default-mode network and dorsal attention network, (corrected by dividing with average brain connectivity). Boxplots show median and quartiles, dot with whiskers represent mean and standard deviation. (b) Functional connectivity between the default-mode network and ventral attention network, (corrected by dividing with average brain connectivity). Boxplots show median and quartiles, dot with whiskers represent mean and standard deviation.

There were no differences between responders and non-responders in baseline cortical GMV (731.3 (70.8) vs. 739.94), white matter volume (WMV; 736.78 vs. 731.45), deep grey matter volume (DGMV; 57.79 vs. 55.72) and lesion volume (13.11 vs. 9.83). In addition, no differences were seen for DTI-based measures of extent (1157.9 vs. 1643.5) and severity (−0.17 vs. −0.32) of white matter damage (Tables 1 and 4). Responders had a significantly lower FC between DMN-VAN compared with non-responders (0.87 vs. 0.98, *p* = 0.018) (Figure 5).

**Table 4.** Microstructural white matter damage and functional connectivity in responders, non-responders and healthy controls.

	Healthy controls (*n* = 21)	Responders (*n* = 22)		Non-responders (*n* = 36)	
		*T*_0_	*T*_1_	*T*_0_	*T*_1_
White matter damage					
Severity (*z*-score)	0.0 (0.23)^†^	–0.17 (0.32)	–0.24 (0.27)	–0.32 (0.35)	–0.28 (0.42)
Extent (number of voxels exceeding *z* = −3.1)	6.67 (14.82)^†^	1157.9 (1699.0)	1459.3 (882.1)	1643.5 (2542.9)	1805.5 (3119.1)
Functional connectivity					
Default-mode network – dorsal attention network	0.81 (0.14)^†^	0.84 (0.12)*	0.84 (0.1)*	0.92 (0.15)*	0.92 (0.16)*
Default-mode network – ventral attention network	0.81 (0.13)^†^	0.87 (0.15)*	0.88 (0.14)*	0.98 (0.19)*	0.96 (0.15)*
Default-mode network – frontoparietal network	1.05 (0.22)^†^	1.18 (0.27)	1.17 (0.23)	1.18 (0.23)	1.16 (0.26)
Dorsal attention network – ventral attention network	1.21 (0.23)	1.13 (0.26)	1.12 (0.27)	1.2 (0.26)	1.16 (0.2)
Dorsal attention network – frontoparietal network	1.11 (0.25)	1.13 (0.25)	1.07 (0.25)*	1.25 (0.24)	1.23 (0.28)*
Ventral attention network – frontoparietal network	0.94 (0.16)	1 (0.18)	1.0 (0.19)	1.06 (0.17)	1.0 (0.16)

Measures of DTI-based white matter damage, resting-state fMRI functional connectivity.

† Significant difference between patients with multiple sclerosis and healthy controls at *p* < 0.05.

* Significant group effect (responders vs. non-responders) at *p* < 0.05.

Finally, responders did not show significantly different FC compared with HC, while non-responders showed significantly higher FC between DMN-DAN (0.92 vs. 0.81, *p* = 0.009) and DMN-VAN (0.98 vs. 0.81, *p* = 0.001), compared with both responders and HC (Figure 5).

### Predicting response

The backward logistic regression identified two independent predictors for response: lower FC between DMN-VAN (*p* = 0.004), and lower FC between DMN-FPN (*p* = 0.029). The model accurately predicted 81.8% of non-responders, and 54.5% of responders (Nagelkerke *R*^2^ = 0.25).

## Discussion

The main aim of this study was to gain insight into the effectiveness of computerized attention training in PwMS and into the clinical and MRI-based characteristics that are associated with successful cognitive training response. Therefore, we performed the conventional analysis of pre–post effects of C-Car on a group level, which yielded results and effect sizes similar to those found in previous cognitive training studies.^3^ Unexpectedly, we saw no significant post-intervention improvement in attention. However, patients in the intervention group showed a modest immediate improvement in working memory (standardized mean difference = 0.37, *p* = 0.038) with no sustained effects on cognitive performance at 3 months follow-up, which is in line with previous results^5,31^ and might indicate the need for a longer duration of cognitive training programmes. Interestingly, we observed a long-term effect for fatigue, with patients in the intervention group showing stable fatigue at the 3 month follow-up compared with the increased fatigue of the waiting-list control group, consistent with previous findings of the C-Car intervention in glioma patients.^11^

Comparing responders and non-responders provided important information. Responders and non-responders did not differ in demographic and clinical variables, baseline cognitive performance, nor in the amount of atrophy and microstructural damage. In previous work, lower grey and white matter volume and a relapsing-remitting disease course were predictive of better response.^6^ The fact that we were unable to reproduce these results might be explained by sample differences. Specifically, patients in our study had a higher cortical GM volume and shorter disease duration (13.52 vs. 21.6 years) compared with patients in the study of Fuchs et al.^6^

Interestingly, regarding fMRI findings, non-responders exhibited higher FC between the DMN and attention networks compared with responders and HC. More precisely, responders showed no differences in FC compared with HC, suggesting an intact connectivity (Figure 5). This relationship between alterations in DMN connectivity and treatment response is in line with previous results^8^ and may be explained by the anti-correlation between DMN and attention networks both in tasks and during rest.^32^ The similarity of our results to those of previous studies with different patient cohorts and interventions,^8^ might suggest that our results are generalizable to other cognitive rehabilitation approaches. This should be further investigated in future studies; if it is indeed the case, this could indicate a mechanism that affects response regardless of cognitive domains affected. Perhaps in non-responders, the DMN insufficiently deactivates when needed, and as such shows an increased connectivity with attention networks. Indeed, in healthy individuals, an increased FC between the DMN and attention networks has been related to poorer attention.^30^ In addition, alterations in DMN connectivity and network dynamics are rather common in PwMS and relate to cognitive impairment.^33,34^ As such, it may well be that an intact FC of the DMN is a prerequisite for successful cognitive training response, regardless of the intervention used. In our multivariate prediction model, lower FC between DMN-VAN and between DMN-FPN (i.e. ‘normal FC’) were both identified as predictors of response. This indicates that the fewer deviations there are from HC-like FC, the higher the chance for successful cognitive training.

Consequently, it seems that the timing of cognitive training in PwMS is of utmost importance. One could argue that a mind-set shift from symptom management towards preventive intervention aimed at preserving cognition is needed (i.e. enhancement of network functioning rather than restoring it, since the latter might be impossible).

Our study is not without limitations. The use of a waiting-list control group is not as optimal as an active control condition.^35^ Also, cognitive impairment was not an inclusion criterion. As a result, the group is heterogeneous in terms of cognitive performance at baseline. That being said, 57.3% of all PwMS were impaired (*z* < −2.0*SD*) at baseline on at least one test. Moreover, although the definition of cognitive decline is well-established in the literature, the definition for response is less clear. We thus decided to rely on a conservative statistical approach (reliable change index). Another limitation of our RCI approach is that multiple tests were included in the domains of attention and working memory. Response in at least one test was calculated as response in the cognitive domain, hence making response in working memory and attention perhaps slightly more likely.

To conclude, our results demonstrated a mild-to-moderate overall short-term working memory effect of a computerized attention training for PwMS. Despite the lack of significant improvement in attention on a group level, we demonstrated that by investigating individual responses to treatment almost 40% of PwMS do improve after training, an effect that would have gone unnoticed in group-level statistics. Response seems to depend on a window of opportunity defined by an intact FC between the DMN and attention networks, allowing the brain to be receptive for the effects of cognitive training. Given the heterogeneity of MS progression, disease course and observed differential response to cognitive training,^5^ it is evident that future studies in the field now need to start exploring individualized (selection) approaches to maximize the effectiveness of cognitive training programmes.^6,8^

## Supplemental Material

sj-docx-1-msj-10.1177_13524585221103134 – Supplemental material for A randomized trial predicting response to cognitive rehabilitation in multiple sclerosis: Is there a window of opportunity? Click here for additional data file. Supplemental material, sj-docx-1-msj-10.1177_13524585221103134 for A randomized trial predicting response to cognitive rehabilitation in multiple sclerosis: Is there a window of opportunity? by Stefanos E Prouskas, Menno M Schoonheim, Marijn Huiskamp, Martijn D Steenwijk, Karin Gehring, Frederik Barkhof, Brigit A de Jong, Margriet M Sitskoorn, Jeroen JG Geurts and Hanneke E Hulst in Multiple Sclerosis Journal
