# Supplementary material for: A randomized trial predicting response to cognitive rehabilitation in multiple sclerosis: Is there a window of opportunity?
Source: Mult Scler. 2022 Jun 28;28(13):2124–36. doi: 10.1177/13524585221103134 (PMC9574229; doi:10.1177/13524585221103134)
Supplement: sj-docx-1-msj-10.1177_13524585221103134 – Supplemental material for A randomized trial predicting response to cognitive rehabilitation in multiple sclerosis: Is there a window of opportunity? [file sj-docx-1-msj-10.1177_13524585221103134.docx]

# SUPPLEMENTARY MATERIAL

# MRI protocol

All PwMS underwent brain MRI scanning at baseline (T_0_) and immediately post-intervention (T_1_). HC underwent MRI scanning only at baseline (T_0_). All subjects were scanned on a 1.5T whole-body MR system (Siemens Magnetom Avanto Syngo, Erlangen, Germany), using an eight-channel phased-array head coil. The whole brain-coverage protocol included:

- 3D T1-weighted MPRAGE sequence for tissue-class segmentation (TR=2700ms, TE=5ms, flip angle=8°, resolution=1.3mm×1.3mm×1.3mm, acquisition time=4:50min.).
- A dual-echo 2D turbo spin echo sequence (PD/T2) for white matter lesion segmentation (TR=3160ms, TE=25/86ms, flip angle=150°, resolution=0.98mm×0.98mm×3.5mm, acquisition time=4:14min.).
- Resting-state fMRI acquired using T2-weighted axial gradient echo planar imaging (EPI), (TR=2850ms, TE=60ms, flip angle=90°, 200 volumes, resolution=3.3mm×3.3mm×3.0mm, acquisition time=9:39min.).
- Diffusion tensor imaging (DTI) sequence for white matter integrity assessment, including one volume without directional weighting, i.e. b0, and 60 volumes with non-collinear diffusion gradients, using EPI, b=700s/mm^2^, TR=6800ms, TE=90ms, flip angle=90°, resolution=2.3mm×2.3mm×2.0mm, acquisition time=7:44min.).

All anatomical scans were reviewed by a radiologist for incidental findings according to standard operation procedures approved by the VUmc Medical Ethical Committee.

## Grey matter, white matter, and lesion volumes

Whole-brain, grey and white matter volumes were calculated on the lesion-filled images using SIENAX, following previously published pipelines and deep grey matter volumes were obtained using FIRST (both part of FSL 6.0.2, https://fsl.fmrib.ox.ac.uk/fsl/fslwiki/). Volumes of individual deep grey matter structures (nucleus accumbens, amygdala, caudate nucleus, hippocampus, pallidum, putamen, thalamus) were normalized for head size using the V-scaling factor of SIENAX and summed to obtain a whole-brain measure of deep grey matter volume.

## Diffusion-weighted Imaging Processing

All pre-processing steps were performed using FSL, including motion- and eddy-current correction on images and gradient-vectors, followed by diffusion tensor fitting. Fractional anisotropy (FA), was derived for each voxel. Each subject's FA image was used to calculate nonlinear registration parameters to the FMRIB58_FA brain. The registered FA images were averaged into a mean FA image, which was skeletonized for tract-based spatial statistics (TBSS) (1). The skeleton was thresholded at 0.2 to include only WM and used for TBSS statistics in all diffusion parameters. For each subject the maximum FA value perpendicular to each voxel of the skeleton was projected onto the skeleton. After the abovementioned analysis, an individual quantification of severity and extent was performed.

First, average FA values were calculated to investigate the severity of damage. Second, whole-skeleton effect sizes were investigated by converting FA values to Z-scores on a voxel basis. For each skeleton voxel of a patient the average value of the HC group was subtracted, and divided by the HC's standard deviation for that voxel. Then, a whole-skeleton mean (unthresholded) Z-score was calculated for FA, indicating the severity across the entire WM skeleton per subject.

Third, to investigate differences in the extent of relatively severe damage, the individual voxelwise Z-scores (see above) were thresholded at P < 0.001, that is, Z > 3.1 or Z < −3.1 compared to the control group. Afterward, the voxels exceeding this threshold were counted, at Z ≤ −3.1 for FA. These counts were log-transformed to a normal distribution for further analysis.

## Resting-state functional connectivity networks

All resting-state functional connectivity (FC) analyses were performed in subject space. Pre-processing of the fMRI data was performed with FEAT, part of FSL, and consisted of: motion correction, spatial smoothing (5mm full width-at-half-maximum Gaussian kernel), and high-pass filtering (100s cut-off). Subsequently, ICA-AROMA (2) was used to remove any residual motion artefacts from the fMRI data. Resting-state fMRI data was first registered to each 3DT1 scan to determine boundary-based registration parameters, which were inverted. Next, the 3DT1 scan was registered to MNI152 standard space to determine non-linear registration parameters, which were also inverted.

To investigate regional FC patterns, an atlas was derived based on a combination of 210 cortical areas from the Brainnetome atlas (3) and 14 deep grey matter regions from FIRST (see supplementary material). For all regions, the average time series were extracted and imported into Matlab (version R2018b). To obtain FC measures, Pearson correlation coefficients between activity of atlas regions were calculated (absolute values). To correct for subject-specific differences in average brain connectivity, the correlation matrix was divided by each subject’s mean correlation. A data reduction step was then performed by averaging regional connectivity values into network connectivity values.

To investigate regional FC patterns, an atlas was derived based on a combination of 210 cortical areas from the Brainnetome atlas and 14 deep grey matter regions from FIRST. Using the inverted non-linear registration parameters, the Brainnetome atlas was co-registered to each participant’s 3DT1 image and masked for grey matter using SIENAX-derived grey matter masks. This atlas was then co-registered to fMRI space using the inverted boundary-based parameters after which only those regions were included that showed at least 30% of voxels remaining after exclusion of less reliable fMRI signal based on a previously reported robust-range approach. Based on these criteria, seven regions were excluded, comprised of bilateral orbitofrontal and inferior temporal areas, and the right nucleus accumbens. The final atlas therefore segmented the fMRI sequence into 217 grey matter regions of which mean time series were obtained.

Thirty-eight out of 224 Brainnetome atlas regions were considered to make up the default-mode network, 24 regions for the dorsal attention network, 20 for the ventral attention network, and 18 for the fronto-parietal network Next, we used a hypothesis-driven approach to define which network connectivity values were further examined, based on their involvement in MS and relevance for attention (4, 5), i.e. the ventral- and dorsal attention networks as well as the fronto-parietal and default-mode networks. Thirty-eight out of 224 Brainnetome atlas regions were considered to make up the default-mode network, 24 regions for the dorsal attention network, 20 for the ventral attention network, and 18 for the fronto-parietal network (Fig. 1). Normally, these networks form a strongly interconnected subnetwork, where the default-mode and attention networks are anti-correlated, both in task and rest (6). The dorsal ventral attention (also known as salience network) networks are known to be involved in top-down and bottom-up attention shifting and stimulus detection, while the fronto-parietal network seems to be involved in the attentional selection of sensory input (7, 8).

# References

1. Smith SM, Jenkinson M, Johansen-Berg H, Rueckert D, Nichols TE, Mackay CE, et al. Tract-based spatial statistics: voxelwise analysis of multi-subject diffusion data. Neuroimage. 2006;31(4):1487-505.

2. Pruim RHR, Mennes M, van Rooij D, Llera A, Buitelaar JK, Beckmann CF. ICA-AROMA: A robust ICA-based strategy for removing motion artifacts from fMRI data. NeuroImage. 2015;112:267-77.

3. Fan L, Li H, Zhuo J, Zhang Y, Wang J, Chen L, et al. The Human Brainnetome Atlas: A New Brain Atlas Based on Connectional Architecture. Cerebral Cortex. 2016;26(8):3508-26.

4. Poole VN, Robinson ME, Singleton O, DeGutis J, Milberg WP, McGlinchey RE, et al. Intrinsic functional connectivity predicts individual differences in distractibility. Neuropsychologia. 2016;86:176-82.

5. Huang M, Zhou F, Wu L, Wang B, Wan H, Li F, et al. Synchronization within, and interactions between, the default mode and dorsal attention networks in relapsing-remitting multiple sclerosis. Neuropsychiatric disease and treatment. 2018;14:1241-52.

6. Raichle ME. The brain's default mode network. Annual review of neuroscience. 2015;38:433-47.

7. Ptak R. The Frontoparietal Attention Network of the Human Brain: Action, Saliency, and a Priority Map of the Environment. The Neuroscientist. 2011;18(5):502-15.

8. Ahrens MM, Veniero D, Freund IM, Harvey M, Thut G. Both dorsal and ventral attention network nodes are implicated in exogenously driven visuospatial anticipation. Cortex; a journal devoted to the study of the nervous system and behavior. 2019;117:168-81.

**Table S1.** Indices used as outcome measures for each neuropsychological test.

| **Neuropsychological test** | **Outcome measure used** |
| --- | --- |
| California Verbal Learning Test – II | Total sum of correct immediate recall trials |
| Letter Digit Substitution Test | 90 seconds correctly read items |
| WAIS-III Digit Span | Digit Span forward total sum |
|  | Digit Span backward total sum |
| WAIS-III Number Letter Sequencing | Total sum of correct items |
| Word List Generation | Mean number of correct animals and professions |
| Location Learning Test | Total sum of correct immediate recall trials |
| Stroop | Colour-word card time minus colour card time |
| D2-test | Total sum correct |
| Concept Shifting Test | Shifting score |
| Test of Everyday Attention | Elevator counting with distraction Score |

**Table S2.** Raw scores on cognitive assessment battery and questionnaires at baseline.

|  | **Healthy controls (*n*=21)** | **MS**  **(*n*=82)** |
| --- | --- | --- |
| **Baseline cognition** |  |  |
| California Verbal Learning Test | **67.24 (7.65)^*^** | 56.44 (11.9) |
| Letter Digit Substitution Test | **64.43 (11.64)^*^** | 55.23 (12.68) |
| Digit Span Forward | **9.71 (1.98)^*^** | 8.67 (2.08) |
| Digit Span Backward | **7.86 (1.28)^*^** | 6.68 (1.8) |
| Number Letter Sequencing | 11.24 (1.87) | 10.48 (2.68) |
| Word List Generation | **17.45 (4.53)^*^** | 15.26 (4.08) |
| Location Learning Test^a^ | 15.1 (10.77) | 22.04 (25.1) |
| Stroop^a^ | 25.86 (7.55) | 30.1 (14.87) |
| D2-test | **156.43 (43.44)^*^** | 129.19 (46.15) |
| Concept Shifting Test^a^ | **8.8 (4.84)^*^** | 12.58 (13.63) |
| Test of Everyday Attention^b^ | **-0.01 (0.60)^*^** | -0.68 (1.08) |
| Average of all tests^b^ | **0.0 (0.51)**^*^ | -0.68 (1.0) |
| **Questionnaires at baseline** |  |  |
| HADS depression^a^ | **2.4 (2.98)^*^** | 5.72 (3.74) |
| HADS anxiety^a^ | **4.8 (4.19)^*^** | 7.78 (4.24) |
| CIS20-r (fatigue)^a^ | **50.85 (25.23)^*^** | 82.32 (20.74) |
| AIS (sleeping difficulty)^a^ | **2.89 (3.13)^*^** | 5.37 (3.63) |
| Coping styles |  |  |
| Active | 19.6 (2.74) | 19.52 (4.34) |
| Seeking distraction | 17.8 (3.16) | 17.72 (4.04) |
| Avoiding | 16.35 (2.76) | 17.32 (3.74) |
| Social support | 15.1 (3.46) | 13.28 (3.83) |
| Passive coping | **10.4 (2.87)^*^** | 12.21 (3.37) |
| Expressing emotions | 5.55 (0.76) | 5.99 (1.64) |
| Reassuring thoughts | 12.45 (1.93) | 12.87 (3.03) |
| Subjective cognition |  |  |
| Cognitive Failures Questionnaire^a^ | **21.35 (11.86)^*^** | 42.7 (19.39) |
| Cognitive Functioning Scale^a^ | **5 (4.26)^*^** | 12.39 (6.04) |

All values represent means and SD, unless otherwise denoted. HADS=Hospital Anxiety and Depression Scale; CIS20-r=Checklist Individual Strength 20-revised. AIS=Athens Insomnia Scale.
a Higher scores indicate worse outcomes.
b Z-score.
* Significant difference between patients with multiple sclerosis and healthy controls at *P*<0.05.

**Table S3.** Raw scores on cognitive assessment battery and questionnaires for responders, non-responders, and healthy controls.

|  | **Healthy controls (*n*=21)** |  | **Responders (*n*=22)** |  |  | **Non-responders (*n*=36)** |  |
| --- | --- | --- | --- | --- | --- | --- | --- |
|  |  | **T_0_** | **T_1_** | **T_2_** | **T_0_** | **T_1_** | **T_2_** |
| **Cognition** |  |  |  |  |  |  |  |
| California Verbal Learning Test | **67.24 (7.65)^*^** | 54.32 (11.63) | 59.77 (12.32) | 60.07 (14.22) | 57.81 (11.37) | 59.44 (11.88) | 60.57 (12.07) |
| Letter Digit Substitution Test | **64.43 (11.64)^*^** | 53.18 (12.4) | 57.59 (15.94) | 58.2 (19.44) | 56.53 (11.22) | 58.28 (11.77) | 58.79 (13.11) |
| Digit Span Forward | **9.71 (1.98)^*^** | 8.41 (1.84) | 9.45 (1.99) | 9.67 (1.95) | 8.58 (2.41) | 8.94 (2.15) | 9.07 (2.19) |
| Digit Span Backward | **7.86 (1.28)^*^** | 6.64 (1.87) | 7.41 (1.76) | 7.4 (1.96) | 6.75 (1.86) | 7.25 (1.99) | 6.86 (2.52) |
| Number Letter Sequencing | 11.24 (1.87) | 9.41 (2.58) | 11.5 (2.52) | 10.53 (3.87) | 10.81 (2.66) | 10.86 (3.16) | 10.36 (3.26) |
| Word List Generation | **17.45 (4.53)^*^** | 15.18 (4.04) | 17.03 (4.42) | 16.38 (5.09) | 15.18 (4.14) | 16.06 (4.46) | 16.77 (3.5) |
| Location Learning Test^a^ | 15.1 (10.77) | 20.55 (21.32) | 14.32 (16.86) | 15.93 (23.38) | 22.49 (26.63) | 16.28 (17.83) | 12.29 (19.52) |
| Stroop^a^ | 25.86 (7.55) | 28.08 (12.5) | 26.69 (11.91) | 26.1 (14.48) | 32.29 (16.75) | 27.95 (13.72) | 27.97 (11.63) |
| D2-test | **156.43 (43.44)^*^** | 122.14 (36.95) | 138.91 (38.85) | 149.07 (45.43) | 129.67 (42.27) | 140.8 (46.16) | 153.82 (51.12) |
| Concept Shifting Test^a^ | **8.8 (4.84)^*^** | 15.8 (18.79) | 9.74 (6.86) | 11.27 (11.67) | 11.21 (8.06) | 9.74 (6.14) | 9.71 (5.46) |
| Test of Everyday Attention^b^ | **-0.01 (0.60)^*^** | -0.79 (1.09) | -0.42 (1.06) | -0.45 (1.05) | -0.64 (0.98) | -0.31 (0.87) | -0.4 (0.99) |
| **Questionnaires** |  |  |  |  |  |  |  |
| HADS depression^a^ | **2.4 (2.98)^*^** | 6.23 (2.94) | 6.76 (4.35) | 7.54 (4.43) | 5.75 (4.17) | 6.53 (3.86) | 7.96 (3.87) |
| HADS anxiety^a^ | **4.8 (4.19)^*^** | 8.23 (4.14) | 4.9 (3.32) | 6.69 (3.84) | 7.33 (4.1) | 5.09 (3.51) | 6.58 (3.53) |
| CIS20-r (fatigue)^a^ | **50.85 (25.23)^*^** | 85.14 (15.04) | 77.14 (19.8) | 80 (17.67) | 81.47 (21.99) | 77.82 (20.59) | 78.96 (19.08) |
| AIS (sleeping difficulty)^a^ | **2.89 (3.13)^*^** | 5.73 (3.18) | 5.67 (3.69) | 4.92 (3.28) | 5.03 (4.18) | 4.81 (4.51) | 4.73 (3.89) |
| Coping styles |  |  |  |  |  |  |  |
| Active | 19.6 (2.74) | 19.77 (4.36) | 20.52 (4.43) | 18.69 (5.12) | 19.19 (4.17) | 18.71 (3.8) | 19.31 (3.4) |
| Seeking distraction | 17.8 (3.16) | 17.5 (4.84) | 17.52 (4.17) | 16.31 (3.92) | 17.64 (3.39) | 16.74 (3.84) | 16.65 (2.94) |
| Avoiding | 16.35 (2.76) | 17.91 (2.99) | 17.1 (3.74) | 18.46 (3.41) | 16.53 (3.68) | 16.32 (3.51) | 16.12 (3.57) |
| Social support | 15.1 (3.46) | 13.36 (3.26) | 13.29 (3.29) | 12.46 (3.89) | 13.25 (4.02) | 13.15 (3.63) | 13.73 (3.85) |
| Passive coping | **10.4 (2.87)^*^** | 12 (2.74) | 11.81 (2.75) | 12.54 (4.12) | 12.06 (3.29) | 11.94 (3.24) | 11.27 (2.96) |
| Expressing emotions | 5.55 (0.76) | 5.59 (1.79) | 5.29 (1.85) | 5 (1.68) | 5.97 (1.59) | 5.38 (1.81) | 5.27 (1.51) |
| Reassuring thoughts | 12.45 (1.93) | 12.68 (3.27) | 13.48 (3.04) | 12.85 (2.88) | 12.53 (2.74) | 11.79 (2.91) | 12.46 (2.97) |
| Subjective cognition |  |  |  |  |  |  |  |
| Cognitive Failures Questionnaire^a^ | **21.35 (11.86)^*^** | 47.09 (16.05) | 40.65 (14.74) | 36.77 (14.71) | 38.63 (18.74) | 36.88 (16.98) | 36.15 (15.62) |
| Cognitive Functioning Scale^a^ | **5 (4.26)^*^** | 13.23 (4.78) | 11.33 (5.04) | 12 (5.76) | 11.08 (5.57) | 11 (5.53) | 11.31 (8.8) |

All values represent means and SD, unless otherwise denoted. HADS=Hospital Anxiety and Depression Scale; CIS20-r=Checklist Individual Strength 20-revised. AIS=Athens Insomnia Scale.
^a^ Higher scores indicate worse outcomes. ^b^ Z-score. * Significant difference between patients with multiple sclerosis and healthy controls at *P*<0.05.
